# Evaluation of CML TKI Induced Cardiovascular Toxicity and Development of Potential Rescue Strategies in a Zebrafish Model

**DOI:** 10.3389/fphar.2021.740529

**Published:** 2021-10-18

**Authors:** Shan Cheng, Pan Jin, Heying Li, Duanqing Pei, Xiaodong Shu

**Affiliations:** ^1^ School of Life Science, Westlake University, Hangzhou, China; ^2^ CAS Key Laboratory of Regenerative Biology, Guangdong Provincial Key Laboratory of Stem Cell and Regenerative Medicine, Guangzhou Institutes of Biomedicine and Health, Chinese Academy of Sciences, Guangzhou, China; ^3^ Joint School of Life Sciences, Guangzhou Institutes of Biomedicine and Health, Chinese Academy of Sciences, Guangzhou Medical University, Guangzhou, China; ^4^ Centre for Regenerative Medicine and Health, Hong Kong Institute of Science and Innovation, Chinese Academy of Sciences, Hong Kong, Hong Kong, SAR China; ^5^ Guangzhou Regenerative Medicine and Health Guangdong Laboratory (GRMH-GDL), Guangzhou, China

**Keywords:** CML, TKI, ponatinib, HQP1351, cardiovascular toxicity, zebrafish

## Abstract

Tyrosine kinase inhibitors (TKIs) to BCR-ABL1 have been successfully used to treat chronic myeloid leukemia (CML), however, multiple TKI-associated adverse events have been reported and become an emerging problem in patients. The mechanisms of TKI-induced toxicity are not fully understood and it remains challenging to predict potential cardiovascular toxicity of a compound. In this study, we established a zebrafish model to evaluate potential *in vivo* cardiovascular toxicity of TKIs. We treated the endothelium labeled *Tg(kdrl:EGFP)* transgenic zebrafish embryos with TKIs then performed confocal imaging to evaluate their vascular structure and function. We found that among FDA approved CML TKIs, ponatinib (the only approved TKI that is efficacious to T315I mutation) is the most toxic one. We then evaluated safety profiles of several clinical stage kinase inhibitors that can target T315I and found that HQP1351 treatment leads to vasculopathies similar to those induced by ponatinib while the allosteric ABL inhibitor asciminib does not induce noticeable cardiovascular defects, indicating it could be a promising therapeutic reagent for patients with T315I mutation. We then performed proof-of-principle study to rescue those TKI-induced cardiovascular toxicities and found that, among commonly used anti-hypertensive drugs, angiotensin receptor blockers such as azilsartan and valsartan are able to reduce ponatinib or HQP1351 induced cardiovascular toxicities. Together, this study establishes a zebrafish model that can be useful to evaluate cardiovascular toxicity of TKIs as well as to develop strategies to minimize TKI-induced adverse events.

## Introduction

The generation of a constitutively active BCR-ABL1 fusion tyrosine kinase due to chromosome translocation is a major cause of chronic myeloid leukemia (CML) and the development of imatinib, the first BCR-ABL1 tyrosine kinase inhibitor, revolutionized the treatment of CML. Second-generation TKIs (nilotinib, dasatinib and bosutinib) and third-generation TKI (ponatinib) were then developed to overcome drug resistance or intolerance (Rossari et al., 2018; Braun et al., 2020). While being very effective in controlling CML, these drugs induce moderate to severe adverse events in patients, especially in the case of ponatinib (Moslehi and Deininger, 2015; Valent et al., 2017; Singh et al., 2020; Iurlo et al., 2021). Indeed, ponatinib’s approval was once suspended by U.S. Food and Drug Administration (FDA) due to serious vascular occlusive events. However, since it is the only currently approved TKI that can inhibit T315I mutant of ABL1, ponatinib was reintroduced as a second-line drug for patients resistant and/or intolerant to prior TKI therapy with a black box warning for vascular occlusion, heart failure and hepatotoxicity.

Additional compounds that are efficacious against T315I are under active development to meet this unsatisfied clinical demand (Rossari et al., 2018). Among these preclinical stage compounds, HQP1351 (previously known as GZD824) is highly potent to T315I mutant of BAC-ABL1 as well as FGFR, PDGFR, FLT3, KIT and is able to inhibit imatinib resistant leukemia or gastrointestinal stromal tumors in animal models (Ren et al., 2013; Liu et al., 2019; Wang et al., 2020b). In addition to all those above mentioned ATP-binding site inhibitors, an allosteric inhibitor that binds to the myristate pocket of BCR-ABL1 and keeps the kinase in an inactive state has been recently developed (Schoepfer et al., 2018). This inhibitor is also efficacious to T315I mutation and less off-target effect is expected. The *in vivo* safety profiles of these clinical stage compounds remain to be established.

Among approved CML TKIs, dasatinib induces pulmonary arterial hypertension (PAH), nilotinib is associated with arterial hypertension and thrombosis, ponatinib induces vascular occlusion events while imatinib and bosutinib appear not cardiovascular toxic (Moslehi and Deininger, 2015; Santoro et al., 2021). While the exact mechanism often remains to be investigated, TKI-induced vascular adverse events is generally believed to be induced by off-target effects such as targeting the VEGF signaling pathway (Agarwal et al., 2018). Biochemical studies of target spectrum indicates that imatinib is the most specific CML TKI and it does not inhibit VEGFR signaling; on the other hand, ponatinib has the broadest substrate spectrum as it inhibits FGFRs, VEGFRs, PDGFRs, KIT, SRC, TIE2, etc. in addition to ABL ((Moslehi and Deininger, 2015; Zeng and Schmaier, 2020; Lee et al., 2021). The ability to suppress VEGFR signaling is consistent with its potential cardiovascular toxicity in ponatinib, however, such correlation does not exist in other TKIs. For example, bosutinib inhibits VEGFRs but it does not induce cardiovascular adverse events, meanwhile, nilotinib and dasatinib do not inhibit VEGFRs but they are cardiovascular toxic. Thus, there must be additional mechanism for TKI-induced cardiovascular adverse events.

While direct targets of TKIs remain to be established, TKI-induced downstream signaling events have been investigated in endothelial cells and animal models. For example, several studies reported that ponatinib treatment in primary human umbilical vein endothelial cells (HUVEC) reduces cell proliferation and viability, inhibits cell migration and disrupts tube formation *in vitro* (Gover-Proaktor et al., 2017; Ai et al., 2018; Haguet et al., 2020), and inhibition of VEGFR2 or activation of Notch-1 signaling pathway is suggested to mediate ponatinib-induced vascular toxicity (Gover-Proaktor et al., 2017; Madonna et al., 2020). In mice, ponatinib induces thrombotic microangiopathy through the VWF-mediated platelet adhesion (Latifi et al., 2019). The vascular toxicity of ponatinib was also observed in a zebrafish cerebral ischemic stroke model where ponatinib is reported to induce vascular endothelial injury, thrombosis, inflammation and impaired animal motility (Zhu et al., 2020). The cardiotoxicity of ponatinib has been investigated in zebrafish as well as isolated neonatal rat cardiomyocytes and ponatinib is reported to induce apoptosis of cardiomyocyte by suppressing the prosurvival AKT and ERK signaling (Singh et al., 2019).

In this study, we established a zebrafish model to evaluate the cardiovascular toxicity of TKIs. We identified ponatinib and HQP1351 as the most toxic CML TKIs and revealed that inhibition of SRC contributes to their vascular toxicity. We then performed proof-of-principle study and demonstrated that among the anti-hypertensive drugs, angiotensin II receptor blockers (ARBs) such as azilsartan and valsartan can effectively rescue ponatinib and HQP1351 induced cardiovascular defect. Thus, our zebrafish model can be used to evaluate potential cardiovascular toxicity of TKIs as well as to develop strategies to reduce TKI-induced adverse events.

## Materials and Methods

### Zebrafish Stocks and Chemical Treatment

Zebrafish were raised and handled according to standard protocols (zfin.org). Animal care and experimental protocols were approved by the Guangzhou Institutes of Biomedicine and Health Ethical Committee. Transgenic line *Tg(kdrl:EGFP)* was used in this study. For chemical treatment, embryos were cultured in 12-well plates (15 embryos per well in 2 ml E3 medium) and treated with the indicated compounds from 2 to 4 days post fertilization (dpf) with chorion removed. The maximum dose of each compound (in range 1–30 µM) that does not induce general developmental abnormality was determined in preliminary experiments and used in cardiovascular toxicity studies. DMSO was used as negative control. Treated embryos were examined at 4 dpf for morphology, circulation and blood vessel patterning. At least two biological repeats were performed and analyzed for each compound. The following compounds were used in this study: imatinib (Targetmol, Boston, MA, T6230), nilotinib (Targetmol, T1524), dasatinib (Targetmol, T1448), bosutinib (Targetmol, T0152), ponatinib (Targetmol, T2372), HQP1351 (Targetmol, T3071), asciminib (Targetmol, T5177), Src-IN-1 (Targetmol, T13061), infigratinib (Targetmol, T1975), AT9283 (Targetmol, T3068), danusertib (Targetmol, T2094), rebastinib (Targetmol, T2640), KW-2449 (Selleck, Houston, TX, S2158), tozasertib (Targetmol, T2509), azilsartan medoxomil (Targetmol, T6219), valsartan (Targetmol, T6716), captopril (Targetmol, T1462), benazepril (Selleck, S5938), amlodipine (Targetmol, T1385), acebutolol (Selleck, S4010), macitentan (Selleck, S8051), metolazone (Selleck, S1610), O-Dianisidine (Sigma, St. Louis, MO, 33430).

### Imaging

Live photos or videos of zebrafish embryos were taken with the Zeiss Axio Zoom. V16 stereo zoom microscope (Zeiss, Oberkochen, Germany). For confocal imaging of zebrafish blood vessels, fixed embryos or cross-sections were imaged with the Zeiss LSM 710 NLO inverted confocal microscope (Zeiss).

### Cryosection

Zebrafish embryos were fixed in 4% PFA/PBS overnight at 4°C, washed three times with PBS then embedded in tissue freezing medium (Leica, Wetzlar, Germany, 14020108926). 20 μm-transverse sections were prepared using a cryotome (Leica, CM3050S). Sections were washed with PBS to remove optimal cutting temperature compound (OCT) and ready for confocal imaging.

### Transmission Electron Microscopy

Zebrafish embryos were fixed in 2.5% (v/v) glutaraldehyde and 2% (v/v) PFA overnight at 4°C, washed in 0.1 M PBS (pH 7.4) and post-fixed in 1% OsO_4_ at RT for 1.5 h. Following gradient ethanol (50, 70, 80, 90, and 100%) and acetone dehydration, samples were embedded in Epon 812 and polymerized for 24 h at 40°C plus 24 h at 60°C. Cross sections (100 nm) were prepared with an ultramicrotome (Leica, EM UC7). Samples were stained with uranyl acetate and lead citrate then examined and photographed with the Tecnai G2 Spirit TEM (FEI).

### Cerebral Thrombosis Assessment

Dechorionated wild type embryos cultured in 12-well plates (15 embryos per well in 2 ml E3 medium) were treated with ponatinib (3 µM), HQP1351 (1 µM) or asciminib (20 µM) from 2 to 4 dpf. For rescue experiments, azilsartan (30 µM) or valsartan (30 µM) was added at the beginning of TKI treatment. DMSO was used as negative control. Embryos were collected at 4 dpf and stained in dark for 15 min in staining solution [o-dianisidine, 0.6 mg/ml, 10 mM sodium acetate, 0.65% H_2_O_2_, and 40% (vol/vol) ethanol]. Stained embryos were cleared with DMSO 3 times then photographed with a stereo microscope (Lecia M205 FA). Experiments were repeated twice and at least fifteen embryos from each group were examined in each experiment.

## Results

### Evaluation of Potential Cardiovascular Toxicity for all Approved CML TKIs in Zebrafish

**FIGURE 1:**
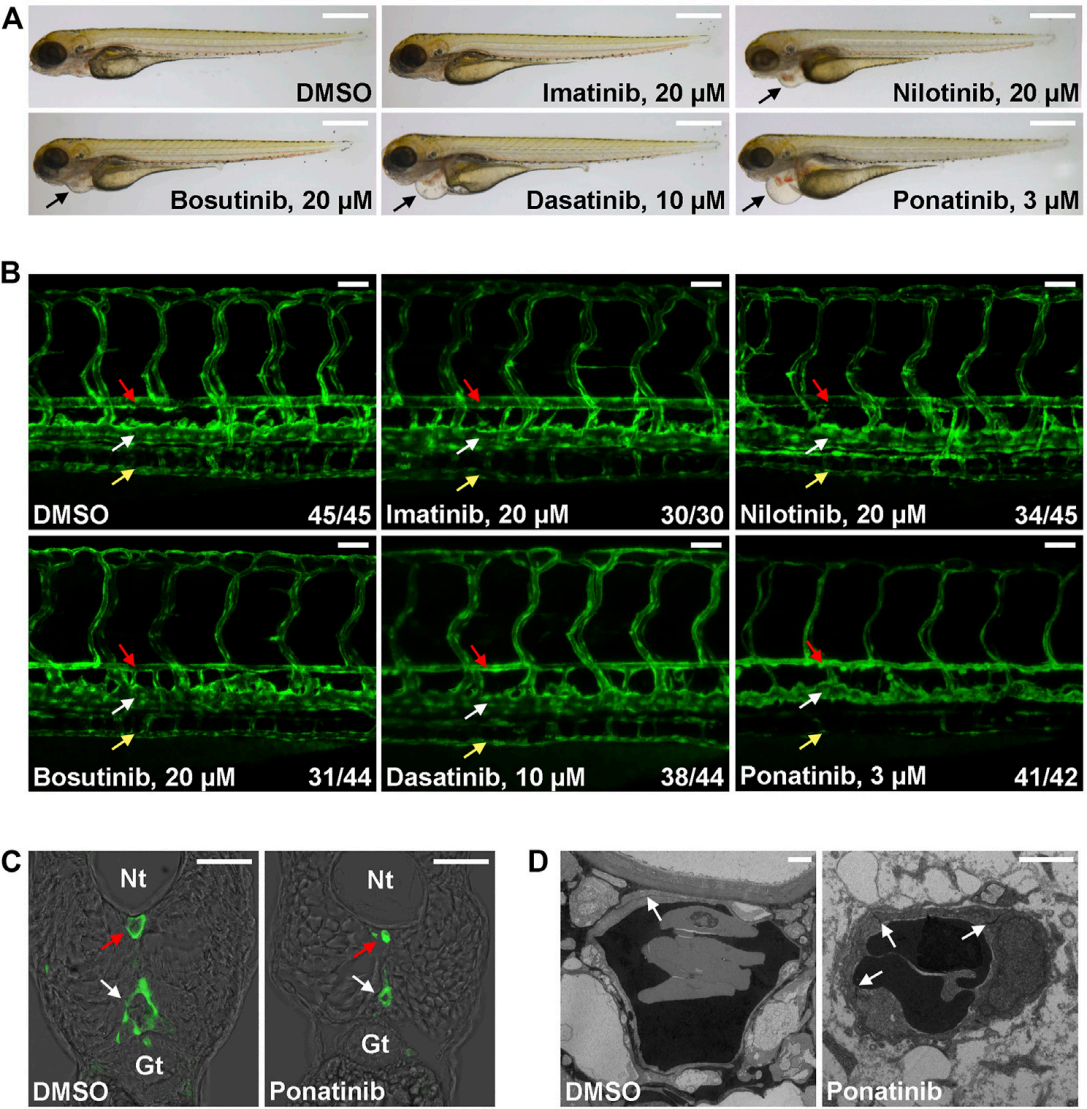
Cardiovascular defects in CML TKI treated zebrafish embryos. **(A)** Live imaging of embryos treated with the indicated concentrations of TKIs from 2 to 4 dpf. Arrows indicate cardiac edema. Scale bar: 500 µm. **(B)** Confocal imaging of vessel defects in TKI treated *Tg(flk:GFP)* embryos. Red arrow: dorsal aorta; white arrow: posterior cardinal vein; yellow arrow: subintestinal vein. At least two biological repeats were performed. Numbers indicate (number of embryos with shown phenotype/total number of embryos analyzed). Scale bar: 50 µm. **(C)** Cross-section of ponatinib-treated embryos. Nt: neural tube; Gt: gut; red arrow: dorsal aorta; white arrow: posterior cardinal vein. Scale bar: 50 µm. **(D)** TEM analysis of ponatinib-treated embryos. White arrow: aortic endothelial cell junctions. Scale bar: 2 µm.

The *Tg(kdrl:EGFP)* is a zebrafish transgenic report line that expresses GFP in endothelial cells and it is widely used in studies related to vessel development and function. We tested whether CML TKI treatment in this transgenic fish line induces cardiovascular defects. Embryos were treated with the listed concentration of TKI at 2 dpf (Figure 1), a time point when major trunk vessels (dorsal aorta, posterior cardinal vein) and intersegmental vessels have formed and active circulation has been established, and embryonic morphology and vasculature were examined at 4 dpf. We found that embryos treated with 20 µM imatinib have normal morphology, vasculature and blood circulation (Figure 1 and Supplementary Videos S1, S2). 20 µM of nilotinib treatment leads to mild cardiac edema (Figure 1A) and mildly reduced circulation (Supplementary Video S3) while the truck/intersegmental vessels appear normal (Figure 1B). In addition to cardiac edema, bosutinib (20 µM) or dasatinib (10 µM) treatment results in stenosis of dorsal aorta (red arrow in Figure 1B) while posterior cardinal vein (white arrow in Figure 1B) and intersegmental vessels are less affected. Circulation is either reduced (Bosutinib, Supplementary Video S4) or barely detectable (Dasatinib, Supplementary Video S5) in the treated embryos. We found ponatinib to be the most toxic CML TKI in this assay. Ponatinib as low as 3 µM induces severe cardiac edema (Figure 1A) and constriction of dorsal aorta and posterior cardinal vein, inhibits the formation of subintestinal vein (yellow arrow in Figure 1B) and no circulation is detectable in the treated embryos (Supplementary Video S6). The constriction of both trunk vessels was confirmed in cross-sections (Figure 1C). We then performed transmission electron microscope analysis to further characterize ponatinib-induced vascular defects. In control embryos, a thin layer of squamous endothelial cells with well-formed cell junctions (white arrow in Figure 1D) is observed. In ponatinib treated embryos, endothelial cells collapse and cell junctions are disrupted to form a thicker and irregular lining layer of vessel. Together, these data indicate that ponatinib disrupts endothelial lining which then leads to vessel constriction in zebrafish embryos.

### SRC Inhibition Contributes to Vascular Toxicity of CML TKIs

**FIGURE 2:**
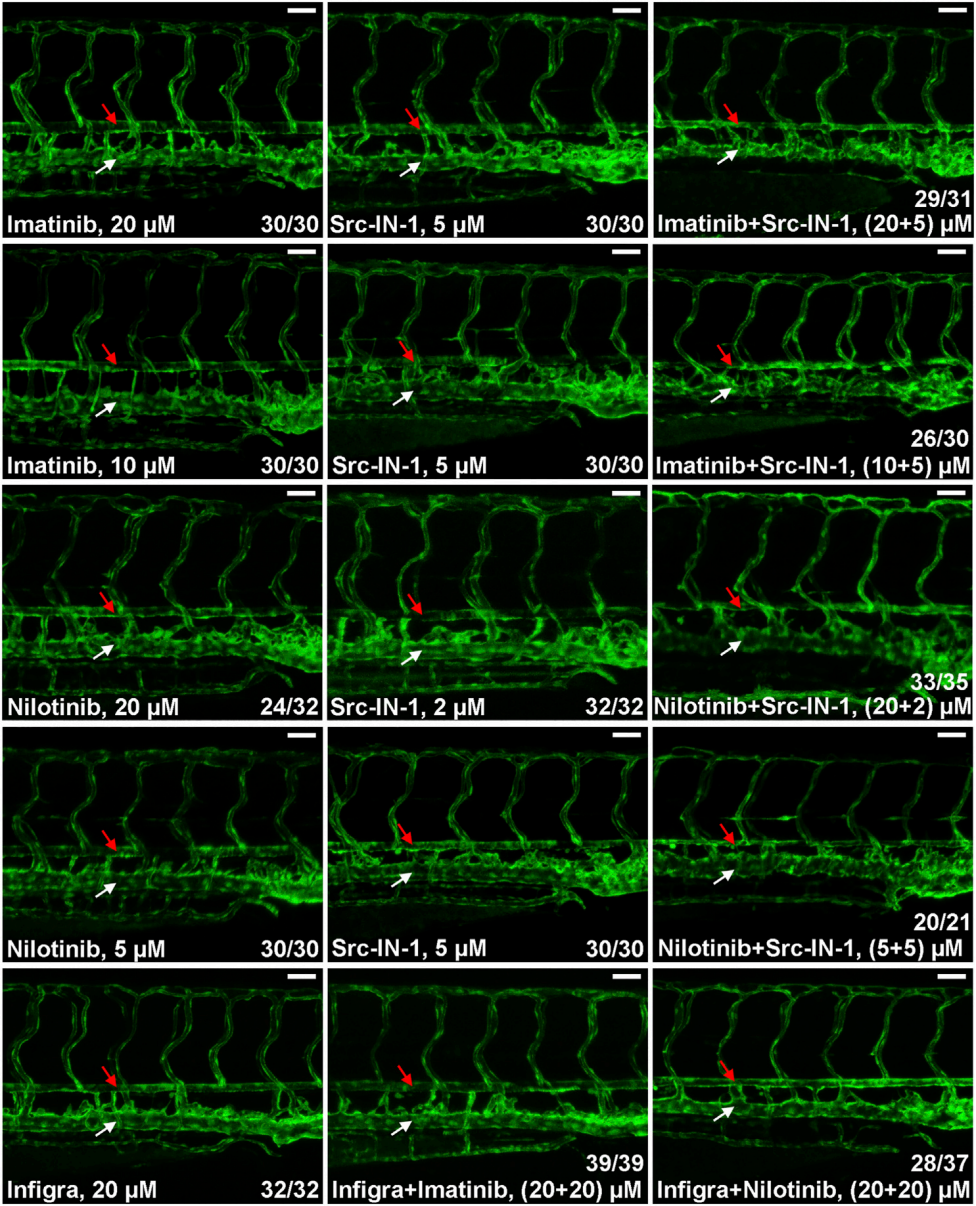
SRC inhibition contributes to stenosis of dorsal aorta. Embryos were treated with the indicated drugs or drug combinations and analyzed as described in Figure 1B. Src-IN-1 is a SRC/LCK inhibitor and infigratinib (Infigra) is a FGFR inhibitor. Combinatory treatment of Src-IN-1 but not infigratinib with imatinib or nilotinib effectively induces stenosis of dorsal aorta (red arrow) while these compounds alone is not sufficient to induce vessel defects. Assays were repeated twice and the list numbers are the sum of two experiments. Scale bar: 50 µm.

In addition to BCR-ABL1, CML TKIs have distinct inhibitory activities to other kinases and the differences in substrate spectrum might contribute to their various cardiovascular toxicities. Previous studies reveal that SRC kinase and FGFRs are common targets for ponatinib, dasatinib and bosutinib but they are not inhibited by imatinib and nilotinib (Moslehi and Deininger, 2015; Rossari et al., 2018; Zeng and Schmaier, 2020; Lee et al., 2021) which is consistent with our observation that ponatinib, dasatinib, bosutinib but not imatinib and nilotinib induce dorsal aorta stenosis in zebrafish (Figure 1B), so we hypothesized that inhibition of SRC or FGFRs is involved in the vasculopathies of CML TKIs. We investigated this hypothesis by testing the effect of SRC inhibitor (Src-IN-1) or FGFR inhibitor (infigratinib) on vessel integrity. We found that Src-IN-1 treatment (5 µM) does not induce stenosis of dorsal aorta, however, a combination of Src-IN-1 (5 µM) with imatinib either at 10 or 20 µM clearly induces stenosis of dorsal aorta (Figure 2). Similarly, combination of Src-IN-1 with nilotinib (2 µM + 20 µM or 5 µM + 5 µM) is able to induce dorsal aorta constriction (Figure 2). On the other hand, infigratinib (20 µM), either alone or in combination with imatinib or nilotinib, is not sufficient to induce constriction of dorsal aorta (Figure 2), indicating inhibition of FGFRs may not be involved in vessel toxicity induced by CML TKIs. Src-IN-1 is known to inhibit SRC or LCK. As *lck* is a T-cell specific gene and it is absent from vasculature from 2 to 4 dpf, the vascular effect of Src-IN-1 is most likely due to the inhibitor of SRC. These results suggest that a combinatory inhibition of ABL1 and SRC may phenocopy ponatinib-induced vascular defects.

### Evaluation of Potential Cardiovascular Toxicity of Clinical Stage Kinase Inhibitors to T315I Mutant

**FIGURE 3:**
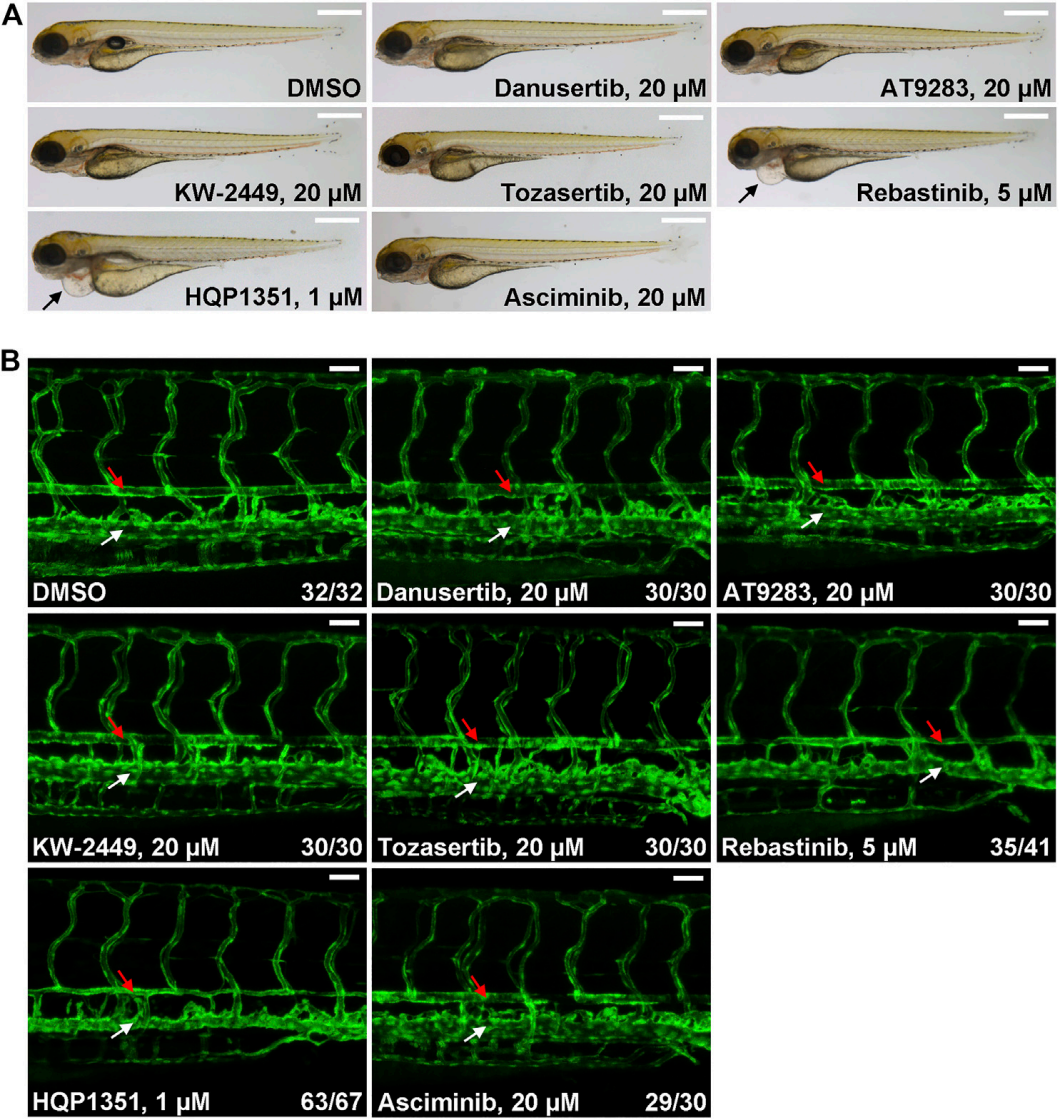
Evaluation of potential cardiovascular toxicity of clinical stage TKIs that are efficacious against T315I. **(A)** Morphology of embryos treated with the indicated TKIs at 4 dpf. Assays were performed as described in Figure 1A. Arrows indicate cardiac edema. Scale bar: 500 µm. **(B)** Confocal imaging of vessel phenotypes of embryos treated with the indicated TKIs. Embryos were treated and analyzed as describe in Figure 1B. Red arrow: dorsal aorta; white arrow: posterior cardinal vein. Numbers are the sum of two independent biological repeats. Scale bar: 50 µm.

Ponatinib is the only approved third generation TKI that can overcome T315I mutation but prominent cardiovascular toxicity limits its application. To meet this unsatisfied medical need, other small chemical inhibitors that can target T315I mutation have been developed while their potential cardiovascular toxicity remains to be investigated. So we used our zebrafish model to evaluate potential cardiovascular toxicities of several clinical stage TKIs. We found that up to 20 µM of danusertib (Gontarewicz and Brummendorf, 2010), AT9283 (Tanaka et al., 2010), KW-2449 (Shiotsu et al., 2009) or tozasertib (MK-0457) (Giles et al., 2013) treated embryos have normal morphology and blood vessel architecture (Figures 3A,B). On the other hand, 5 µM of rebastinib (DCC-2036) (Eide et al., 2011) induces cardiac edema and stenosis of dorsal aorta, indicating potential cardiovascular toxicity. HQP1351, which shares similar structure and inhibitory repertoire with ponatinib (Ren et al., 2013), induces severe cardiovascular toxicity: HQP1351 at 1 µM is able to induce severe cardiac edema and stenosis of dorsal aorta (Figures 3A,B) which is the most toxic one among all TKIs we examined. Asciminib treatment (up to 20 µM) does not lead to noticeable cardiovascular toxicity (Figures 3A,B). These data reveal dramatically different cardiovascular toxicities of those T315I targeting TKIs in zebrafish model and suggest that precaution should be taken during further development of those TKIs with clear vasculopathies.

### Proof-of-Principle Study to Rescue Ponatinib/HQP1351 Induced Cardiovascular Toxicities

**FIGURE 4:**
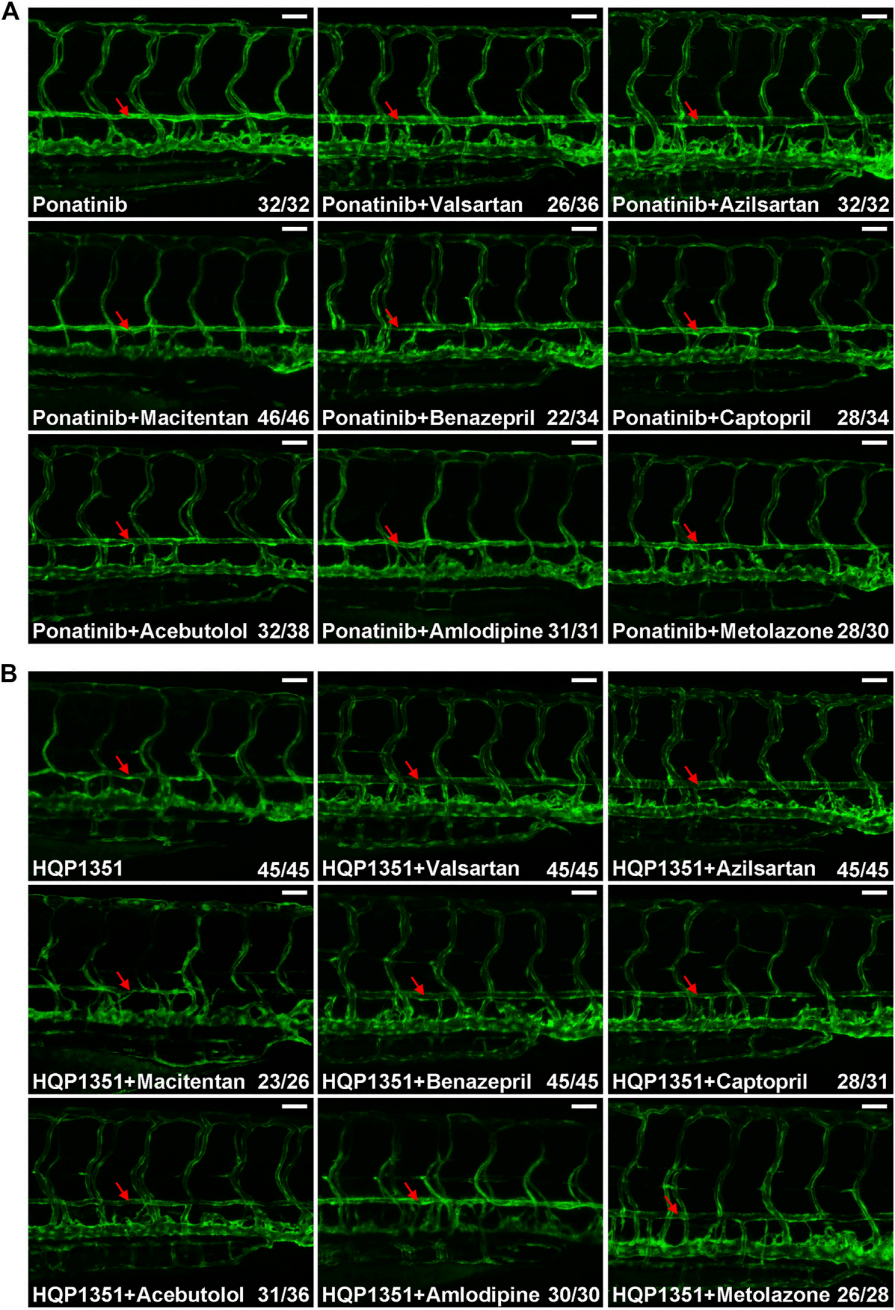
Effects of anti-hypertensive drugs on ponatinib or HQP1351 induced dorsal aorta constriction. **(A)** Embryos were co-treated with the indicated compounds from 2 to 4 dpf and analyzed as in Figure 1B. Valsartan and azilsartan are ARBs and able to rescue ponatinib-induced dorsal aorta constriction. Other anti-hypertensive drugs are less efficacious to minimize ponatinib-induced vascular defects. **(B)** Valsartan, azilsartan, benazepril and metolazone can rescue HQP1351-induced vascular constriction while macitentan, captopril, acebutolol and amlodipine cannot. Assays were repeated and analyzed as described in Figure 1B. Red arrow: dorsal aorta. Scale bar: 50 µm.

Small chemicals are widely used in zebrafish studies to modify development or physiology of embryos. So we performed proof-of principle studies to investigate whether or not ponatinib-induced vascular constriction can be rescued by small chemical compounds. We first tested commonly used anti-hypertensive drugs since many of them have vasodilation function. Embryos were treated with ponatinib (3 µM) together with the indicated anti-hypertensive drugs at 2 dpf and the vascular morphology and circulation of treated embryos were examined at 4 dpf. We found ARBs such as valsartan (30 µM) and azilsartan (30 µM) fully rescue ponatinib-induced vascular stenosis (Figure 4A) and improve circulation (Supplementary Videos S7, S8). On the other hand, endothelin receptor antagonist (macitentan, 5 µM), angiotensin-converting enzyme (ACE) inhibitors (benazepril and captopril, 30 µM), beta blocker (acebutolol, 30 µM), calcium channel blocker (amlodipine, 30 µM) and diuretic (metolazone, 30 µM) are not effective in rescuing ponatinib-induced cardiovascular toxicity (Figure 4A) and defective circulation (Supplementary Videos S9–S14). We then tested whether these compounds can rescue HQP1351 induced cardiovascular toxicity. We found that valsartan and azilsartan are effective in rescuing HQP1351 induced vascular constriction and circulation defects (Figure 4B and Supplementary Video S15). In addition, we found that the ACE inhibitor benazepril and the diuretic metolazone are able to reverse HQP351 induced vascular defects while they fail to do so in the majority of ponatinib treated embryos. Macitentan, captopril, acebutolol and amlodipine fail to rescue both ponatinib and HQP1351 induced vascular defects (Figure 4B).

**FIGURE 5:**
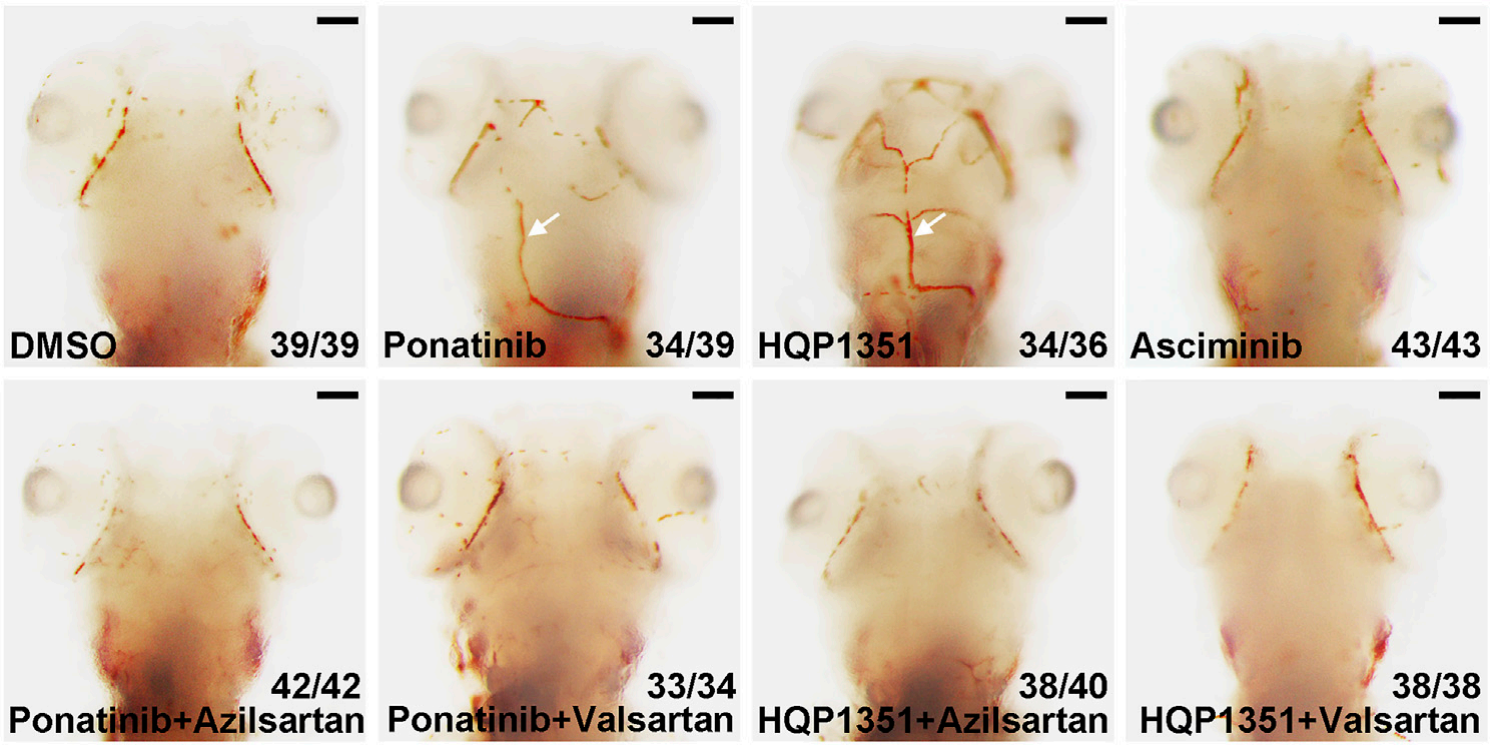
Azilsartan and valsartan rescue ponatinib or HQP1351 induced cerebral thrombosis. Embryos were treated with the indicated drugs from 2 to 4 dpf and stained with o-dianisidine to visualize cerebral thrombosis. Ponatinib (3 µM) or HQP1351 (1 µM) induces cerebral thrombosis (white arrows) while asciminib (20 µM) does not induce this defect. Ponatinib or HQP1351 induced cerebral thrombosis can be prevented by co-treatment of embryos with either azilsartan (30 µM) or valsartan (30 µM). Assays were repeated twice and the list numbers are the combined results. Scale bar: 100 µm.

Ponatinib is recently reported to induce cerebral thrombosis in a zebrafish ischemic stroke model (Zhu et al., 2020). We found that HQP1351 (1 µM) but not asciminib (20 µM) also induces cerebral thrombosis in the same assay (Figure 5) which is consistent with their different activities to induce vessel stenosis. Since azilsartan and valsartan can rescue ponatinib or HQP1351 induced dorsal aorta stenosis and restore trunk circulation, we analyzed whether they are able to rescue cerebral thrombosis as well. We found that co-treatment with azilsartan (30 µM) or valsartan (30 µM) fully rescues ponatinib (3 µM) or HQP1351 (1 µM) induced cerebral thrombosis (Figure 5). Together, these results suggest that ponatinib/HQP131 may induce vessel constriction and cerebral thrombosis via over-activation of the angiotensin signaling pathway and ARBs could have clinical value in preventing ponatinib induced vascular occlusive events in patients.

## Discussion

Vasculopathies have been documented among VEGFR or CML TKIs while their underlying mechanisms remain largely uncertain. Thus, it is difficult to evaluate or predict potential vascular toxicities for candidate compounds in early stages of drug development. In this study, we established a robust zebrafish model to evaluate cardiovascular toxicity of TKIs. In this model, imatinib does not show any noticeable toxicity while ponatinib and dasatinib induce severe cardiovascular defect, which is consistent with their observed cardiovascular toxicity in patients. Toxicity of bosutinib is observed only at very high concentration (20 µM) which is unlikely to occur in patients. Nilotinib (20 µM) induces cardiac edema and reduced circulation, however, the vasculature appears normal in treated embryos. These results suggest the underlying mechanism for cardiovascular toxicity might be different between nilotinib and ponatinib.

Inhibition of VEGF receptor signaling is well known to induce vasculopathies, however, it might not be the cause of CML TKI induced vessel defects. Bosutinib inhibits VEGFRs but it does not demonstrate vascular toxicity in patients while the vascular toxic dasatinib does not inhibit VEGFRs (Moslehi and Deininger, 2015; Zeng and Schmaier, 2020; Lee et al., 2021). We revealed that inhibition of SRC contributes to vascular toxicity as combinatory inhibition of ABL1 and SRC is sufficient to induce vessel constriction. Rebastinib and HQP1351 are two clinical stage BCR-ABL1 inhibitors that also inhibit SRC and we found both of them induce vessel stenosis, further supporting a critical role of SRC in vessel integrity. A possible role of SRC in zebrafish vessel integrity is supported by other published studies as well. For example, PP1 is an inhibitor of SRC family kinases as well as BCR-ABL TKI (Hanke et al., 1996; Tatton et al., 2003) and it has been reported to induce dorsal aorta defect similar to that induced by ponatinib (Zhong et al., 2011). The authors reported that inhibition of SRC by a small chemical inhibitor or morpholino mediated inhibition of SRC does not induce vascular constriction which is consistent with our Src-IN-1 data (Figure 2). These results indicate that SRC inhibition alone is not sufficient to induce vascular toxicity but it cannot be deduced that TKI-induced vascular toxicity is SRC-independent. Indeed, we found that co-treatment of embryos with imatinib and SRC inhibitor induces vascular toxicity similar to those induced by ponatinib, indicating an important role of SRC in vascular maintenance. Interestingly, ibrutinib which is a BTK inhibitor that also inhibits SRC (Bye et al., 2017) is recently reported to induce constriction of zebrafish dorsal aorta (Wang et al., 2020a). Together, these observations suggest that SRC inhibition is associated with potential vascular toxicity in certain TKIs and zebrafish could be used as a robust and convenient *in vivo* model to evaluate potential cardiovascular toxicity of candidate compounds during early stage of drug development.

Ponatinib is currently the only approved TKI that can target T315I mutation of ABL1, however, multiple adverse events including cardiovascular toxicities occur after long term exposure to ponatinib which limits its usage. HQP1351, which is another third generation CML TKI, is under active development as an alternative to ponatinib. HQP1351 shares similar chemical structure with ponatinib and we show here that HQP1351 and ponatinib induce similar cardiovascular toxicity in zebrafish. This result indicates that HQP1351 might share common cardiovascular toxicity with ponatinib in patients as well. So patients should be carefully evaluated for potential cardiovascular adverse events and constantly monitored during drug exposure. Recently, a novel allosteric inhibitor of ABL1 named asciminib is developed. Instead of binding to the ATP-binding pocket, asciminib binds to the myristoyl binding pocket specific to ABL1 and locks the enzyme in a catalytically inactive conformation. Asciminib is efficacious against T315I and expected to induce less off-target effect. We report here that asciminib does not induce noticeable vascular toxicity and cerebral thrombosis. Together with a recent report that asciminib is less cardiotoxic (Singh et al., 2019), these results suggest that asciminib has better cardiovascular safety profile than ponatinib or HQP1315 and it could be a promising TKI for CML with T315I mutation.

Chemical screens have been successfully carried out in zebrafish model for various purposes. We performed proof-of-principle rescue screen to reduce ponatinib/HQP1351 induced cardiovascular toxicity and discovery that commonly available anti-hypertensive drugs including ARBs such as valsartan and azilsartan are able to rescue ponatinib or HQP1351 induced cardiovascular toxicity. Interestingly, in a recent cohort analysis in CML patients, it is reported that inhibition of the renin-angiotensin system (RAS) by ACE inhibitors or ARBs is associated with reduced arterial occlusive events in hypertensive CML patients treated with second or third generation BCR-ABL inhibitors (Mulas et al., 2020). Our study provide direct experimental support for the observation and the zebrafish model can be used to investigate the underlying mechanism for those TKI induced cardiovascular toxicities. In addition to regulate blood pressure, RAS signaling in bone marrow is established to regulate multiple steps of hematopoiesis or leukemogenesis in an autocrine and/or paracrine manner (Haznedaroglu and Malkan, 2016). Targeting this local RAS signaling may reduce the production of neoplastic cells in leukemia. For example, the expression of RAS components is elevated in CML patients and imatinib treatment is able to reduce RAS expression (Sayitoglu et al., 2009). Together, these studies suggest that targeting RAS in CML patients may block neoplastic cell production as well as minimize TKI-induced cardiovascular toxicity. Thus, RAS inhibition could be an adjunctive therapy during TKI targeted treatment of CML. Potential benefits and risks of this strategy should be investigated in future clinical studies.

## Data Availability

The original contributions presented in the study are included in the article/Supplementary Material, further inquiries can be directed to the corresponding authors.
